# Toward Stabilizing the Keyhole in Laser Spot Welding of Aluminum: Numerical Analysis

**DOI:** 10.3390/ma17194741

**Published:** 2024-09-27

**Authors:** Saeid SaediArdahaei, Xuan-Tan Pham

**Affiliations:** Department of Mechanical Engineering, École de Technologie Supérieure, 1100, Notre-Dame Ouest Street, Montreal, QC H3C 1K3, Canada; saeid.saediardahaei.1@ens.etsmtl.ca

**Keywords:** keyhole, instability analysis, velocity, curvature effect, Marangoni effect, Darcy’s damping force

## Abstract

The inherent instability of laser welding, particularly keyhole instability, poses significant challenges in industrial applications, leading to defects such as porosities that compromise weld quality. Various forces act on the keyhole and molten pool during laser welding, influencing process stability. These forces are categorized into those promoting keyhole opening and penetration (e.g., recoil pressure) and those promoting keyhole collapse (e.g., surface tension, Darcy’s damping forces), increasing instability and defect likelihood. This paper provides a comprehensive instability analysis to uncover key factors affecting keyhole and process instability, presenting future avenues for improving laser welding stability. Using a novel numerical method for simulating laser spot welding on aluminum with COMSOL Multiphysics 5.6, we investigated the effect of laser pulse shaping on keyhole and process instability. Our analysis focused on keyhole morphology, fluid flow behaviour, and force analysis. The results indicated that the curvature effect, Marangoni effect, and Darcy’s damping force are primary contributors to instability, with the curvature effect and Darcy’s damping force being the most dominant. Additionally, erratic and high-velocity magnitudes induce intense fluid flow behaviour, exacerbating keyhole instability. Moreover, single/quadruple peak triangular and variant rectangular ramp-down pulse shapes produced the least instability, while multi-pulse rectangular shapes exhibited intense instability. It was found that combining triangular/rectangular pulse shapes can reduce force and keyhole instability by smoothing spontaneous force spikes, resulting in a more stabilized welding process. Controlling fluid flow and abrupt force changes with appropriate pulse shaping is key to defect-free welded products.

## 1. Introduction

**Figure 1:**
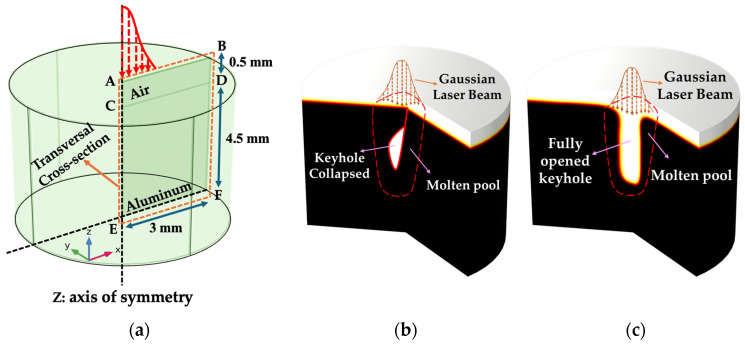
Schematic 3D representation of (**a**) the 2D axisymmetric laser welding setup with Gaussian beam profile, (**b**) laser-based welding process with keyhole collapse (closed keyhole) induced by instabilities, and (**c**) laser-based welding process with keyhole stability (open keyhole).

Laser welding is widely employed in automobile and aerospace manufacturing due to its ability to deeply penetrate materials, particularly aluminum alloys, which possess reliable characteristics such as lightweight, good corrosion resistance, and high strength as highlighted by Miller et al. [1], Wang et al. [2], and Schubert et al. [3], just to name a few. The fundamental processes occurring during laser–material interaction in high-energy-density laser processing encompass a range of intricate physical phenomena. These include dynamics related to surface tension, the generation of recoil pressure from vaporization, the formation of vapour plumes, attenuation and scattering of the laser beam by condensed plume materials, phase transitions such as melting, solidification, and vaporization, thermo-capillary driven flows (Marangoni convection and curvature effects), flow induced by recoil pressure, multiple reflections of the incident laser beam, and high-speed rates of heating and cooling [4,5,6]. During laser welding, a high-intensity laser beam creates a ‘keyhole’ by heating the material above its vaporization point. This causes vaporization and mass loss, forming a hole. The resulting vapour pressure, or recoil pressure, helps penetrate the material and keep the keyhole open [7]. Keyhole formation and stability throughout the laser welding process are influenced by various forces, including recoil pressure, surface tension (Marangoni and curvature effects), buoyancy, hydrostatic pressures, and gravity. These forces cause fluctuations in the keyhole and affect its propagation. Among them, recoil pressure and surface tension emerge as dominant factors, with recoil pressure opening the keyhole and surface tension attempting to close it [8]. D. Schauer et al. [9] emphasized the importance of surface tension and recoil pressure on the keyhole wall. They identified a critical equilibrium point where these forces balance each other. Below this point, recoil pressure dominates, while above it, surface tension prevails. When surface tension becomes dominant, keyhole stability is compromised, potentially leading to collapse. Figure 1 illustrates a schematic 3D depiction of laser welding, showing how the process instability causes periodic keyhole openings and collapses, as reported by Jiang et al. [10] and Zhang et al. [11]. Understanding this dynamic behaviour and fluctuations in the keyhole, along with the inherent instability in laser welding, has constrained its widespread adoption within the industrial manufacturing sector, as noted by You et al. [12]. Instabilities in laser welding, particularly with aluminum, lead to defects such as porosities [2], humps [13], spatters [14], and other imperfections, significantly undermining the reliability of manufactured products. Most defects in laser welding of aluminum and its alloys mainly arise from aluminum’s material properties [15], its rapid solidification rate [15], process-related instability [12], and high reflectivity [16]. Zhang et al. [17] found that instability in the process directly leads to defect formation and You et al. [12] highlighted that keyhole instability causes bubble formation in aluminum alloys. Huang et al. [15] noted that the rapid solidification rates of molten aluminum trap bubbles at the solidification front, resulting in porosities. Therefore, controlling fluctuations in the keyhole and melt pool is crucial for process stability and aluminum welding quality, as keyhole instabilities are a common cause of porosity, as emphasized by Lin et al. [18]. A variety of experimental methods were employed in scholarly research to understand the laser welding process. Techniques included ex situ X-ray tomography for porosity analysis [19], in situ high-speed imaging of the melt pool [20] and vapour plume [21], and synchrotron-based X-ray imaging to observe melt pool dynamics and keyhole formation [22]. These setups are often costly and complex, with some facing limitations in spatial or temporal resolution, hindering detailed observation of rapid phenomena. In addition, obtaining high-quality welds in high thermal conductivity materials or volatile elements, like aluminum and certain alloys (e.g., 5000 or 7000 series), frequently depends on a trial and error approach. The narrow welding parameter window for these materials further complicates experimentation, as keyhole and weld pool behaviour exhibit erratic fluctuations [23]. Hence, experimentation might fail to reliably identify the causes of keyhole instability, dominant forces, and fluid flow behaviour contributing to process instability and defect formation. Numerical simulations alternatively offer significant advantages over experimental methods for modelling and understanding keyhole dynamics and instabilities, providing a more efficient and cost-effective solution [24]. Pulsed wave (PW) lasers are ideal for welding aluminum and its alloys, as they effectively overcome aluminum’s reflectivity by providing high energy density at the start of the pulse while regulating the average power to reduce overall heat [25,26]. Wang et al. [27] noted that using pulse wave modulation (PWM) in laser beams enhances welding stability. By adjusting laser characteristics like frequency, amplitude, and power, the size and stirring intensity of the molten pool can be effectively controlled. Matasunawa et al. [28] conducted an extensive study on the dynamics of the molten pool and the keyhole, revealing that PWM significantly reduces porosity formation. They attributed this reduction to the efficient elimination of holes from the preceding pulse by the subsequent pulse, given an appropriate overlapping ratio. In another paper, Tsukamoto et al. [29] confirmed that modulating laser power in pulses effectively stabilizes the keyhole and reduces porosity, particularly when the modulation frequency aligns with the natural oscillation frequency of the molten pool. Moreover, Heider et al. [30] studied power modulation when performing laser welding on copper, demonstrating that modulating power and frequency effectively reduced weld defects and increased penetration depth by up to 30%. Volpp et al. [31] also investigated methods to improve the Gaussian beam welding stability and discovered that the laser beam shaping helps keep the keyhole open, preventing its collapse. This highlights the importance of modifying the laser beam shape to ensure welding stability. However, these studies did not provide information on the dominant forces causing instability and defects, nor did they address how modulating laser power affected these forces. To evaluate the effect of laser pulse modulation on the stability of keyhole mode laser welding, numerical models must account for fluid flow dynamics in the melting pool and accurately capture the dynamic evolution of the keyhole, considering heat transfer, fluid motion, and phase transitions. Scientists employed various modelling approaches to address these factors in their simulations. Pang et al. [32] employed the Level Set (LS) method to simulate keyhole formation and investigated the mechanisms of keyhole instability with varying heat inputs. Courtois et al. [5] proposed a detailed laser welding model utilizing the LS method to trace the vapour/liquid interface, encompassing all metal phases. However, using a higher laser wavelength than typical industrial standards raises concerns about its practicality. Moraitis et al. [33] employed the Finite Element Method (FEM) to develop a localized model that predicts keyhole morphology according to temperature profile. The model was based on solid mechanics and excluded fluid convection or the evolution of the free surface. Previous studies primarily focused on reducing defects through various operational and laser parameters but often lacked detailed explanations of the forces and fluid flow behaviours causing instabilities, both quantitatively and conceptually. Furthermore, the impact of pulse modulation on these instabilities and defect reduction was not thoroughly explored. While several studies have explored defect reduction and laser welding in other materials, a significant gap remains in understanding how advanced pulse modulation techniques, particularly non-rectangular and combined pulse shapes, can be used to control keyhole instabilities in aluminum. This research uniquely addresses this gap by offering both quantitative and conceptual new insights that have the potential to optimize laser welding processes and enhance industrial applications in fields such as automotive and aerospace manufacturing. Understanding these aspects is crucial for comprehending how laser–material interactions, molten pool dynamics, and keyhole behaviour contribute to instability, and how these factors can be controlled. Most research concentrated on rectangular pulse shapes and materials like steel and copper, with limited theoretical studies on aluminum. The present study addresses the gap in conducting pulse shaping in keyhole mode laser spot welding on aluminum using various triangular, variant rectangular pulse shapes, and their combinations. The aim was to investigate how pulse shaping can be used to control keyhole instability and improve process stability, and to assess the sensibility of instability on these pulse shapes. A quantitative analysis of instability-inducing forces, fluid flow behaviour, and keyhole morphology was conducted to analyze the instability. This study employed a novel numerical model using modified techniques, including a modified mixture theory, a modified LS method, and the enthalpy–porosity technique, to investigate phase transformations and coupled physics in a 2D axisymmetric configuration. The modified mixture theory was applied comprehensively across liquid, solid, and gas phases, while the modified LS method tracked the vapour/liquid interface and included evaporation effects throughout the laser welding process. The enthalpy–porosity technique enhanced the model’s capability to accurately capture phase transformations and fluid flow dynamics.

## 2. Materials and Methods

This research developed a 2D axisymmetric model of laser spot welding on aluminum to investigate the impact of pulse shaping on keyhole stability and process instabilities. This study analyzed triangular and rectangular pulse shapes and their combinations to improve efficiency and understand the forces and fluid behaviours causing instabilities. The choice of these particular pulse shapes was made based on their potential to reduce instabilities and defects in keyhole mode laser welding, as suggested by preliminary studies [34]. These shapes were chosen to explore the full spectrum of their effects on keyhole instabilities and fluid dynamics through a thorough instability analysis.

**Figure 2:**
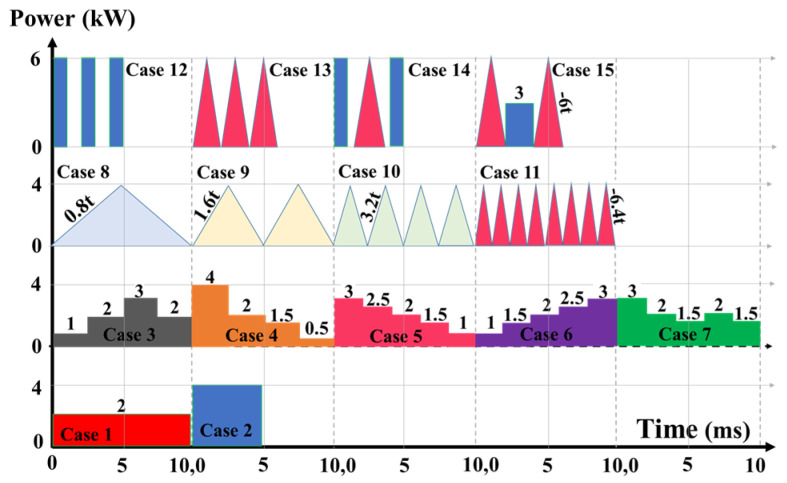
Full schematic illustrating the pulse shapes implemented for pulse modulation.

The goal was to control and reduce these instabilities for defect-free weldments. Case studies based on various pulse shaping trends are detailed in Table 1 and Figure 2.

### 2.1. Model Geometrics, Material, and Laser Heat Source

All simulations and generation of the results were conducted using the COMSOL Multiphysics 5.6 graphical user interface. The software allows an immediate transition from a 2D axisymmetric model to a 3D model and imposes symmetry conditions for axisymmetric assumptions. Aluminum, with dimensions of 4.5 mm (height) × 3 mm (width), was chosen as the base metal, with its thermophysical properties listed in Table 2. Aluminum was selected as the base material due to its widespread use in industrial applications, particularly in the automotive and aerospace industries. Its high thermal conductivity, reflectivity, and challenging weldability make it an ideal candidate for studying the effects of pulse modulation on keyhole stability and understanding the underlying reasons behind keyhole instability through sensibility analysis. A Gaussian laser distribution represented the heat source for stationary laser welding. The 3D schematic of the simulated 2D axisymmetric configuration is shown in Figure 1. This model was used because stationary spot laser welding keeps the laser beam fixed, ensuring rotational symmetry about the vertical z-axis as shown in Figure 1a. This assumption was necessary to validate the numerical model and provided a robust foundation for future enhancements, facilitating the transition to more complex setups, including moving lasers and ensuring alignment with industrial applications.

### 2.2. Heat and Fluid Flow Model

This study used the modified LS method, an Eulerian technique, to capture the behaviour of the free surface and the vapor/liquid interface, and to compute the normal and tangential elements of the free surface. Moreover, previous studies utilized the modified mixture theory to model the simultaneous existence of solid and liquid phases within the mushy zone [35,38]. The current study extended the mixture theory to include all three phases (solid, liquid, and gas) within the whole domain, accounting for molten, vaporized, and solidified material. The mixture properties were defined by volume fractions of solid, liquid, and gas, ensuring an accurate representation of phase transitions and their impact on material behaviour during welding. These methodologies were employed to simulate keyhole formation and molten pool behaviour, taking into account free surface dynamics, recoil pressure, surface tension effects, buoyancy forces, evaporation effects, and mass loss due to vaporization. The numerical investigation was based on the following assumptions:

- The movement of molten material in the fusion zone was simulated assuming Newtonian behaviour, incompressibility, and laminar flow characteristics.
- Temperature-dependent changes in aluminum’s thermophysical properties were disregarded. Instead, the modified mixture theory was applied to calculate thermophysical properties, such as thermal conductivity, specific heat, and density, for each element by utilizing fixed values for the solid, liquid, and gas phases of aluminum. These properties were then averaged according to the phase proportions within each element, yielding an effective constant thermophysical property for the simulation [34].
- The mushy zone was treated as a porous medium permeated by molten metal [35].
- Plasma effects and the Knudsen layer were excluded from the model.
- Multiple reflections of the laser beam within the keyhole were disregarded in this study. This assumption is justified for scenarios with low penetration depths, as multiple reflections become significant primarily in cases of deeper weld penetration (greater than 600 μm) [7]. Moreover, the laser beam coefficient of absorption was assumed constant at keyhole walls.
- The vaporized material was modelled as an ideal gas that is transparent to the incoming laser beam.

The transport phenomena in all three phases (solid, liquid, vapour) present during the welding process were addressed by solving the conservation equations of energy (Equations (1)–(9)), mass conservation (Equation (10)), and momentum (Equations (11)–(15)). Also, the modified conservative equation of the LS method was solved to account for evaporation (Equations (16) and (17)). The governing equations are briefly provided below, with more detailed information available in our previous article on pulse wave modulation [34].

- Energy equation and its concerning parameters

The thermal field calculations were performed by solving the transient heat conservation equations across both the solid/liquid and gas regions:

(1) $$\rho C_{p}\frac{\partial T}{\partial t}+\rho C_{p}\vec{u}.\nabla T=\nabla .\left(k\nabla T\right)+(q_{Laser}-Q_{vapor})\delta \left(\phi \right)$$

(2) $$q_{Laser}=\frac{\alpha 2P_{Laser}}{\pi R_{eff}^{2}}exp(\frac{-2r^{2}}{R_{eff}^{2}})B_{t}$$

(3) $$B_{t}=\left\{\begin{array}{c} 1,\;\;t\leq t_{p}\\ 0,\;\;t>t_{p} \end{array}\right.$$

(4) $$Q_{vapor}=-L_{V}{\dot{m}}_{H-L}$$

(5) $${\dot{m}}_{H-L}=\sqrt{\frac{M}{2\pi R}}\frac{P_{sat}\left(T\right)}{\sqrt{T}}(1-\beta_{r})$$

(6) $$P_{Sat}=P_{atm}\exp\left[\frac{ML_{v}}{RT_{v}}\left(1-\frac{T_{v}}{T}\right)\right]$$

where *T* represents temperature, *t* denotes time, ρ is the density, *C_p_* stands for specific heat capacity, *k* is the thermal conductivity, and $\vec{u}$ is the velocity vector. *q_laser_* and *Q_vapor_* are the heat sources, representing the laser energy and the energy loss by evaporation. α is aluminum’s absorptivity. $P_{Laser}$ represents the peak laser power, and $B_{t}$ refers to the temporal distribution of the laser, applied to simulate the effects of the pulse wave laser welding, respectively. $R_{eff}$ refers to the effective radius of the laser beam spot and $t_{p}$ represents the duration of the pulse. To apply the laser heat flux to the material surface and account for evaporation-induced energy loss, the delta function associated with the LS variable ($\delta \left(\phi \right)$) was used. The energy loss through evaporation was deducted from the laser energy density, and the remaining energy was multiplied by the delta function of the LS variable to apply these effects on the vapour/liquid interface. In addition, ${\dot{m}}_{H-L}$ represents the evaporative mass loss, while $P_{Sat}$ denotes the saturated vapour pressure expressed by the Hertz–Langmuir relation and the Clausius–Clapeyron law, respectively [39]. $\beta_{r}$ stands for the retro-diffusion coefficient, whereas *R* refers to the universal gas constant and *M* represents the molar mass of vaporized particles. The enthalpy of fusion *L_m_* and evaporation *L_V_* were introduced through equivalent specific heat using Equations (7)–(9) [39,40].

(7) $${Cp}_{eff}={Cp}_{sl}+L_{m}D_{m}+L_{V}D_{V}$$

(8a) $$D_{m}=\frac{{exp}^{\left(\frac{-{\left({T-T}_{m}\right)}^{2}}{{{dT}_{m}}^{2}}\right)}}{\sqrt{\pi {{dT}_{m}}^{2}}},$$

(8b) $${T_{m}-dT}_{m}\to {T_{m}+dT}_{m}$$

(9a) $$D_{V}=\frac{{exp}^{\left(\frac{-{\left({T-T}_{V}\right)}^{2}}{{{dT}_{V}}^{2}}\right)}}{\sqrt{\pi {{dT}_{V}}^{2}}},$$

(9b) $${T_{V}-dT}_{V}\to {T_{V}+dT}_{V}$$

The functions $D_{m}$ and $D_{V}$ are Gaussian functions centred around the melting temperature ($T_{m}$) and vaporization temperature ($T_{V}$), respectively. The equivalent specific heat capacity was determined by incorporating the specific heat of the solid/liquid mixture ${Cp}_{sl}$ into the calculation (mixture theory), as outlined in Equation (7). The smoothing range for the melting (${dT}_{m}$) was chosen to be 29 K, based on half the difference between the solidus and liquidus temperatures of the material $\left({(T}_{l}-T_{s})/2\right)$ [39]. For vaporization, the smoothing range (${dT}_{V}$) was selected to be 50 K, as suggested by Tomashchuk et al. [40].

- Modified mass conservation equation and recoil pressure

The impact of recoil pressure was added to the continuity equation using a source term, which was suggested by Zhang et al. [35] and Courtois et al. [41] as presented in Equation (10):

(10) $$\nabla .\vec{u}=\delta \left(\phi \right){\dot{m}}_{H-L}(\frac{\rho_{l}-\rho_{v}}{\rho^{2}})$$

where $\delta \left(\phi \right)\;$introduced a Heaviside function for the LS variable to ensure smooth phase transitions and interface traversal in finite element computations [35]. This method minimized discontinuities, particularly for temperature-sensitive material properties and forces such as surface tension, recoil pressure, and the laser heat source, which are confined to the interface. Multiplying these forces by $\delta \left(\phi \right)$, they were confined to the vapour/liquid interface during calculations, ensuring they only had non-zero values at that precise location.

- Momentum equation:

(11) $$\rho \left(\frac{\partial \vec{u}}{\partial t}+\vec{u}.(\vec{\nabla }.\vec{u})\right)=\vec{\nabla }.[-pI+\mu (\vec{\nabla }\vec{u}+{\left(\vec{\nabla }\vec{u}\right)}^{T})])+\rho \vec{g}-\rho_{l}\beta_{l}(T-T_{melting})\vec{g}\phi -\mu_{l}K\vec{V}+(\gamma .nk-\nabla_{s}\gamma .t)\delta \left(\phi \right)$$

(12) $${\vec{F}}_{st}=\left(\gamma .nk-\nabla_{s}\gamma .t\right)\delta \left(\phi \right)$$

(13) $${\vec{F}}_{Buoyancy=}-\rho_{l}\beta_{l}(T-T_{melting})\vec{g}\phi$$

(14) $$F_{Darcy\;Damping}=-\mu_{l}K\vec{u}$$

(15a) $$K=\frac{\frac{180}{d^{2}}{\left(1-V_{l}\right)}^{2}}{{V_{l}}^{3}+b},$$

(15b) $$V_{l}=\left\{\begin{array}{c} 1,\;T>T_{l}\\ \frac{T-T_{s}}{T_{l}-T_{s}},\;T_{s}\leq T\leq T_{l}\\ 0,T<T_{s} \end{array}\right.$$

where μ is the dynamic viscosity, and ${\left(\vec{\nabla }\vec{u}\right)}^{T}$ indicates the transpose of the gradient of the velocity vector $\vec{u}$. *ρg* represents the effect of gravity, ${\vec{F}}_{st}$ is the surface tension effect, ${\vec{F}}_{Buoyancy}$ is the buoyancy effect with $\beta_{l}$ serving as the liquid’s volume expansion coefficient, and $F_{Darcy\;Damping}$ is Darcy’s damping force. *b* was used to avoid division by zero, *d* is a constant proportional to the dendrite size and set to 10^−2^ cm [42], and $V_{l}$ is the volume fraction of the fluid.

- Modified transport equations of the LS method

The vapour/liquid interface tracing was carried out using the modified LS method, as described in Equation (16). This study introduced an improved conservative Level Set method, combining the Volume of Fluid (VOF) approach with the narrow band LS method and included a gas dynamic source term [43]. This source term accounted for evaporation effects, caused by vapor pressure and mass loss at the interface. The equations were as follows:

(16) $$\frac{\partial \phi }{\partial t}+\vec{u}.\nabla \phi -\delta \left(\phi \right){\dot{m}}_{H-L}\left(\frac{V_{f,1}}{\rho_{v}}+\frac{V_{f,2}}{\rho_{l}}\right)+\gamma_{ls}\nabla .(\phi \left(1-\phi \right)\frac{\nabla \phi }{\left|\nabla \phi \right|}-\epsilon_{ls}\nabla \phi )=0$$

(17) $$\phi \left(x,y,t\right)=\left\{\begin{array}{c} 0,\;{\;\;\;\;\;\;T>T}_{V},\;\;y<{-\epsilon }_{ls}\\ 0.5,\;\;\;T_{l}<T<T_{V},\;\;y=0\\ 1,\;\;\;\;\;\;\;\;\;\;T<T_{l},\;\;y>\epsilon_{ls} \end{array}\right.$$

where $\gamma_{ls}$ refers to the reinitialization parameter associated with the flow velocity, while $\epsilon_{ls}$ regulates the thickness of the interface. Additionally, ϕ is the LS variable that varies smoothly between 0 and 1 within the interface layer, being set to 0.5 at the vapour/liquid interface, as shown in Equation (17). Defining this variable across all elements of the computational domain, and transporting it through fluid flow calculations, facilitated tracking the vapor/liquid interface and distinguishing between condensed and gaseous phases. The third term on the left side of Equation (16) is the source term added to the standard transport equations of the LS equation, enhancing the effect of evaporation-induced mass loss at the interface. This term facilitated the smooth transport of the LS variable$\;\left(\phi \right)$ in finite element calculations on both sides of the interface by using the $\delta \left(\phi \right)$, as well as the volume fractions and densities of the liquid and gaseous phases. Additionally, $V_{f,1}$ represents the volume fraction of gas, and $V_{f,2}$ denotes the volume fraction of a solid/liquid mixture.

- Boundary and initial conditions

As depicted in Figure 1a, a Gaussian laser heat flux is applied to boundary CD, which is also subjected to convective heat transfer to the environment ($h(T_{ext}-T_{a})$) and radiative heat transfer ($\xi K_{b}(T^{4}-{T_{a}}^{4}$), where *K_b_* is the Stefan–Boltzmann constant, and $T_{a}$ is the ambient temperature. The initial temperature was set to 296 K, with the initial velocity and pressure values considered zero at the surface and for all solid surfaces far from the heat source. Boundary AE was designated as the axis of symmetry, ensuring that no flow crosses the axis (AE), and the temperature gradient remains zero along the axis. A wetted wall condition was imposed on boundaries BD, DF, and EF. All other boundaries were treated as thermally insulated.

### 2.3. Numerical Considerations

Melting and solidification were modelled using the thermal enthalpy–porosity technique [35], while evaporation was simulated with the modified conservative LS method [43]. Additionally, the modified mixture theory was employed to account for mixture effects, simplifying finite element calculations at the interface, particularly for the elements containing multiple phases [35,38]. The modified mixture theory, Level Set (LS) method, and enthalpy–porosity technique were employed due to their strong capability to model phase transformations, fluid flow, and keyhole morphology. These methods facilitate a comprehensive understanding of the complex vapour/liquid and solid/liquid interactions, thereby ensuring high fidelity and reliable results. Each of these methods was independently implemented within the LS, heat transfer in fluids, and laminar flow modules of COMSOL Multiphysics. To account for the coupling effects between the incorporated methods and interfaces, two COMSOL Multiphysics coupling interface modules were employed. The LS, mixture properties, and laminar flow were coupled using the two-phase flow interface. Additionally, the non-isothermal Multiphysics interface was used to couple the heat transfer in fluids and laminar flow interfaces. Finally, these two coupling modules were further integrated under the overarching Multiphysics interface within COMSOL to ensure comprehensive calculations.

#### 2.3.1. Numerical Setup

**Figure 3:**
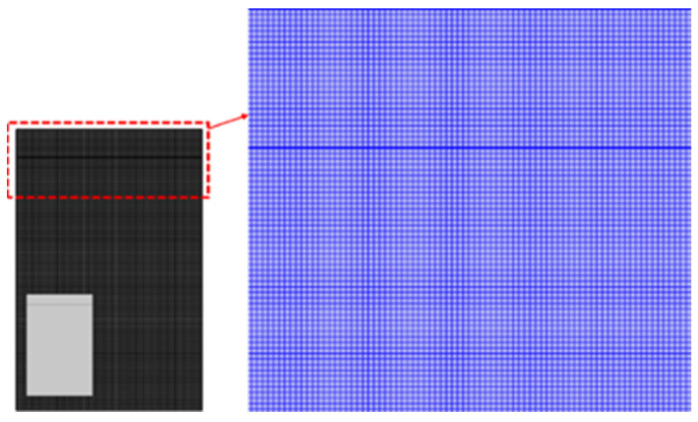
Computational domain with the corresponding extra-fine mapped mesh.

The model was developed in the Multiphysics interface of COMSOL Multiphysics 5.6, using the coupling heat transfer, fluid motion, and transport equations of LS. A mapped mesh with quadrilateral elements and extra-fine meshes was used and calibrated for fluid dynamics (Figure 3). The element size chosen for the mesh was set at 0.02 mm. The simulation, which modelled 10 ms of laser welding over 17 h, was run on a Lenovo ThinkStation P720 workstation, equipped with an Intel^®^ Xenon^®^ Gold 5118 CPU (12 cores, 24 logical processors) with 128 GB RAM. The CPU was manufactured by Intel corporation, headquartered in Santa Clara, CA, USA. The system was assembled by LENOVO, based in Beijing, China. Time steps were set at 10 μs. For fluid flow calculations, a PARDISO direct solver with a nested dissection multithreaded preordering algorithm was used, while the LS transport and heat transfer equations employed a PARDISO direct solver with an automatic preordering algorithm. Before running the simulation, mesh element count, reinitialization parameter, and interface thickness were verified to ensure reliable outcomes. Sensitivity analysis was conducted iteratively, refining parameters by monitoring convergence trends and keyhole depths. Mesh sensitivity analysis involved testing four different mesh counts: 16,968, 24,320, 37,500, and 48,045 elements. The optimal mesh element count was determined to be 37,500, as keyhole morphology and depth showed no significant changes with more elements. The best reinitialization parameter and interface thickness values for the LS technique were found to be 5 m/s and 0.03 mm, respectively, which provided better computational efficiency and convergence. Further variations in these parameters showed no significant impact on keyhole depth and morphology.

#### 2.3.2. Model Validation

**Figure 4:**
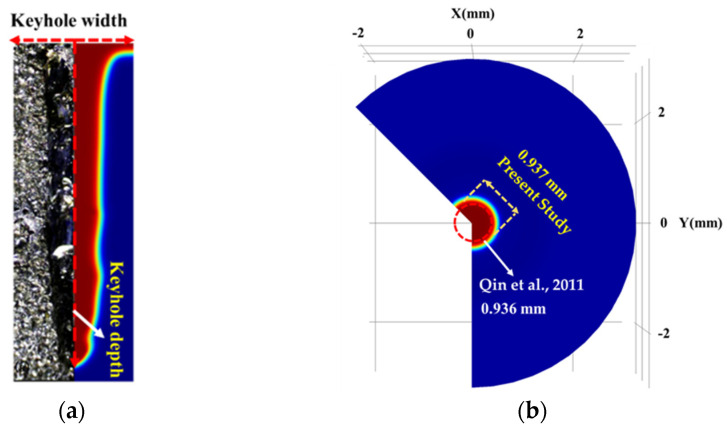
Comparison of (**a**) the keyhole morphology from the simulation with the experimental results taken from Qin et al. [44] and (**b**) the keyhole surface diameter.

The current numerical model was validated with the experimental work of Qin et al. [44] and presented in our previous article [34]. Correspondingly, the keyhole penetration depth, shape, and width obtained from the current numerical model were compared to the observed keyhole morphology from Qin et al.’s experiments [44], as shown in Figure 4. The simulation used the same laser characteristics: total laser energy of 18 J, pulse width of 3 ms, and spot radius of 300 µm with the one utilized by Qin et al. [44]. The numerical results showed a keyhole penetration depth of 3.837 mm, closely matching the experimental value of 3.824 mm. Simulated and experimental keyhole spot diameters on the surface were approximately 0.937 mm and 0.936 mm, respectively. The maximum keyhole width reported experimentally was about 0.407 mm, while the numerical simulations showed a width range of 0.310 mm and 0.51 mm. The model accurately predicted the keyhole depth and diameter, with slight deviations of 6–12% in keyhole width. These discrepancies could be attributed to differences in material properties, the neglect of multiple laser beam reflections, and assumptions about metallic vapour transparency and plasma effects. Despite these factors, the model successfully correlated with the experimental results, effectively predicting keyhole diameter and depth, and providing acceptable keyhole width values.

#### 2.3.3. Instability Analysis Procedure

Increased instability directly correlates with keyhole collapse probability, leading to severe fluctuations, collapses, and defect formation. This study investigates the parameters contributing to keyhole instability by examining three main criteria as follows:

Geometry: keyhole stability is achieved by maintaining an open keyhole with slight fluctuations and a symmetrical shape with smooth transitions from top to bottom. Ensuring these geometrical characteristics reduces the risk of keyhole collapse. Force analysis: a stable keyhole is characterized by the equilibrium between the driving forces acting on the keyhole and the molten pool. Stability and a fully open keyhole are maintained as long as the equilibrium between recoil pressure and the forces contributing to keyhole collapse—such as surface tension, Darcy’s damping force, gravity, and buoyancy—is preserved [8,9]. The sensibility of these driving forces across different cases with various pulse shapes presented in this article was analyzed to understand the impact of fluctuations and magnitudes of these collapse-inducing forces on keyhole instability and overall process stability. Fluid behaviour: high-velocity magnitudes and multiple peaks indicate intense fluid flow behaviour, which might contribute to keyhole instability. Intense fluid flow within the keyhole and molten pool might increase instability due to chaotic fluid motion, leading to irregular keyhole walls and a higher risk of collapse.

## 3. Results and Discussion

This section provides an in-depth analysis of the impact of pulse shaping on keyhole instability and penetration depth. Keyhole instability is examined through detailed assessments of morphology, geometry, quantitative force analysis, and fluid flow behaviour, as discussed in the following sections.

### 3.1. Keyhole Geometry Analysis

**Figure 5:**
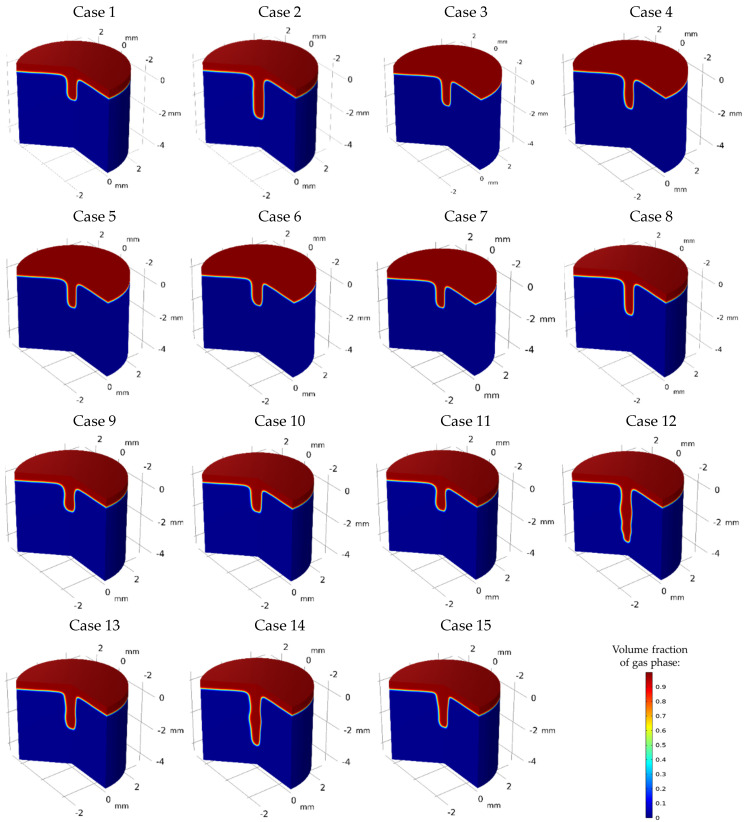
Three-dimensional depiction of keyhole morphology and temporal evolution with various pulse shaping cases.

Keyhole mode laser welding begins with irradiating high laser energy density on the material, raising its temperature. When the material’s temperature exceeds the melting and vaporization points, it undergoes fusion and evaporation, forming and propagating the keyhole due to recoil pressure and mass loss from the surface. Figure 5 shows different keyhole geometries at the end of the welding process, considering various laser energy pulse shapes defined in Table 1 and Figure 2. The penetration depth in Case 2 with PW laser welding was significantly greater than in Case 1 with continuous wave welding (CW), attributable to PW’s higher laser energy density, which accelerated recoil pressure domination and keyhole formation. Moreover, both Cases 1 and 2 exhibited symmetrical keyholes. However, Case 2 demonstrated slightly steeper wall angles at the top, which suggests increased fluctuations, likely due to the use of higher initial laser power. For Cases 3 to 7, variant rectangular pulse shapes with fixed total laser energy of 20 J were analyzed, showing stable and cylindrical keyhole shapes. With higher initial laser power, Case 5, featuring a ramp-down pulse shape, generated a deeper and narrower keyhole compared to Case 6, which had a ramp-up shape. The faster keyhole formation and the dominance of recoil pressure in Case 5 contributed to greater penetration and concentrated energy at the bottom part of the keyhole, rather than dispersing it along the walls to widen the keyhole. Moreover, Case 4 showed a higher potential for keyhole collapse at the top due to steeper wall angles, leading to pinching and potentially causing the keyhole to become narrower and less stable. Additionally, the significant power drop from 4 kW to 2 kW at the second rectangular step promoted solidification and allowed other driving forces to dominate over recoil pressure, closing the keyhole. Simultaneous ramp-down/up pulse shapes, as seen in Cases 3 and 7, resulted in smoother transitions from top to bottom, enhancing stability without significantly affecting penetration depth. Cases 8 to 11 investigated triangular pulse numbers, maintaining total laser energy at 20 J. Increasing from one to eight triangular pulses (single, double, quadruple, and octuple peaks), Cases 8 and 10 exhibited more stable keyholes with smoother transitions from top to bottom. Case 8 showed a narrower and deeper keyhole compared to the wider keyhole in Case 10, and both cases showed no wall fluctuations or inward pulling of the walls in the top part of the keyhole. In contrast, Cases 9 and 11 displayed wall fluctuations and inward pulling of the keyhole walls at the top. Cases 12 to 15 explored higher laser energy densities with shorter pulse periods, reducing total laser energy to 18 J. Despite lower total energy, full penetration depth increased due to higher starting laser powers and accelerated keyhole formation. However, significant wall fluctuations were noted, indicating instability and collapse risk. Case 12 had the highest penetration depth but also the greatest instability. Mixed high-energy rectangular and triangular pulses (Cases 13 and 15) resulted in more stable keyholes, balancing depth and width to reduce collapse risk.

### 3.2. Instability-Inducing Forces: Quantitative Analysis

**Figure 6:**
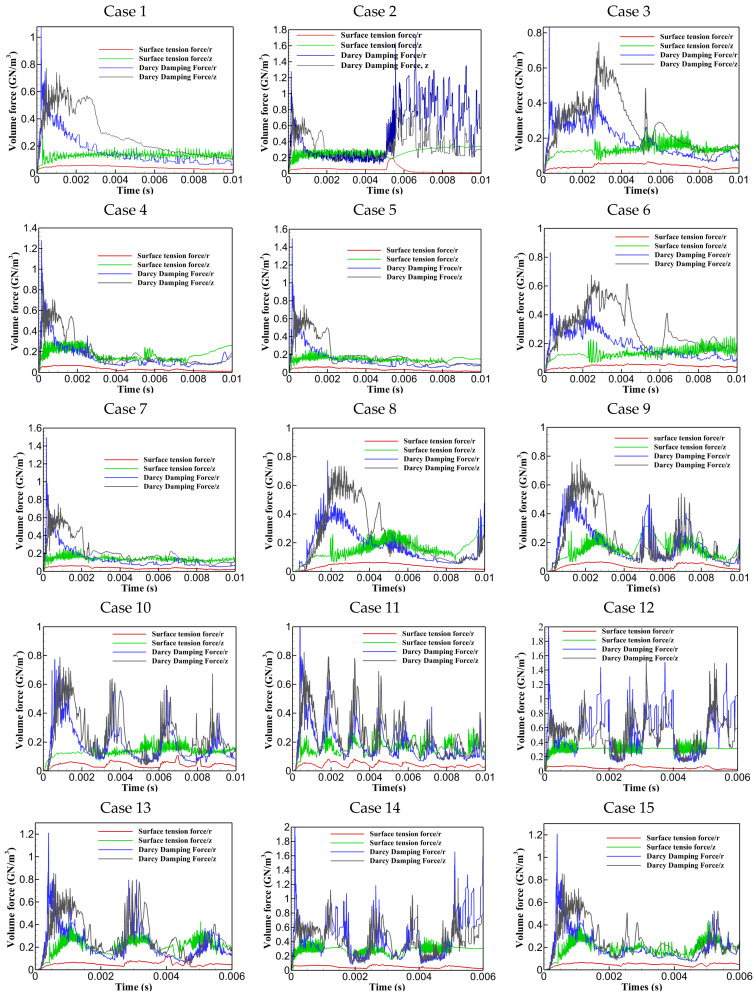
Quantitative force analysis for Cases 1 to 15.

This section presents a quantitative analysis of forces contributing to keyhole collapse and instability, including surface tension forces (curvature effect and Marangoni effect), Darcy’s damping forces, gravity, and buoyancy, as illustrated in Figure 6. It was found that buoyancy and gravity forces had a negligible impact compared to other forces. The surface tension force in the axial direction (curvature effect) was more intense than in the radial direction (Marangoni effect), creating a pressure difference across the keyhole walls that pulled them inward, increasing the likelihood of collapse. The next dominant force was Darcy’s damping force in the axial and radial directions, which contributed to keyhole instability by resisting fluid motion within the mushy zone. At the beginning of the laser pulse, a large amount of energy was abruptly deposited into the material, causing rapid temperature increases, intense fusion, vaporization, and keyhole formation. As vapour pressure rapidly increased due to steep temperature gradients, there was a sharp spike in Darcy’s damping force and surface tension. As the laser continued to interact with the material, the system reached a quasi-steady state where the molten pool and keyhole stabilized to some extent with a fully opened keyhole, causing the forces to equilibrate. According to the diagrams, both the curvature and Marangoni effects are greater for PW compared to CW due to greater laser energy deposition in shorter pulse periods. Additionally, more erratic behaviour was seen for Darcy’s damping force after turning off the laser in PW. In correlation with the keyhole morphology presented in Figure 5, it was found that erratic variations in Darcy’s damping force and greater curvature and Marangoni effects contributed to steeper keyhole wall angles and inward pulling in the upper part of the keyhole, indicating more instability. Analyzing Cases 3 to 7, Case 3, which used a ramp-down varying rectangular pulse shape, exhibited the maximum curvature effect with multiple peaks and erratic behaviour compared to other cases. This was supported by data indicated in Figure 5, showing less keyhole wall stability. The steeper wall angles at the top of the keyhole resulted from the inward pulling due to greater pressure differences associated with the higher curvature effect. Ramp-down varying rectangular pulse shapes (Cases 4, 5, and 7) showed faster force stabilization around and after 0.002 s compared to ramp-up cases (Cases 3 and 6), which exhibited prolonged fluctuations. Cases 5 and 7, with gradual ramp down and gradual ramps of down-up-down, showed fewer fluctuations and faster force stabilization. For Cases 8 to 11, increasing the number of triangular pulse peaks correlated with heightened erratic fluctuations, instabilities in the forces, and multiple peaks. Case 8 demonstrated relatively smoother force variations, which in correlation with its keyhole morphology, contributed to a smoother transition from the top to the bottom of the keyhole, resulting in greater stability. In comparing Cases 12 through 15, it was observed that Cases 12 and 14, utilizing single multi-pulse rectangular and triangular shapes, experienced stronger curvature effects and Darcy’s damping forces than the combined multi-pulse shapes in Cases 13 and 15. This led to increased keyhole instability and greater fluctuations along the keyhole walls. Case 15 showed the least curvature effect and fluctuations in the Marangoni effect, along with the least Darcy’s damping force, all contributing to a more stable and open keyhole, as corroborated in Figure 5.

### 3.3. Fluid Behavior

**Figure 7:**
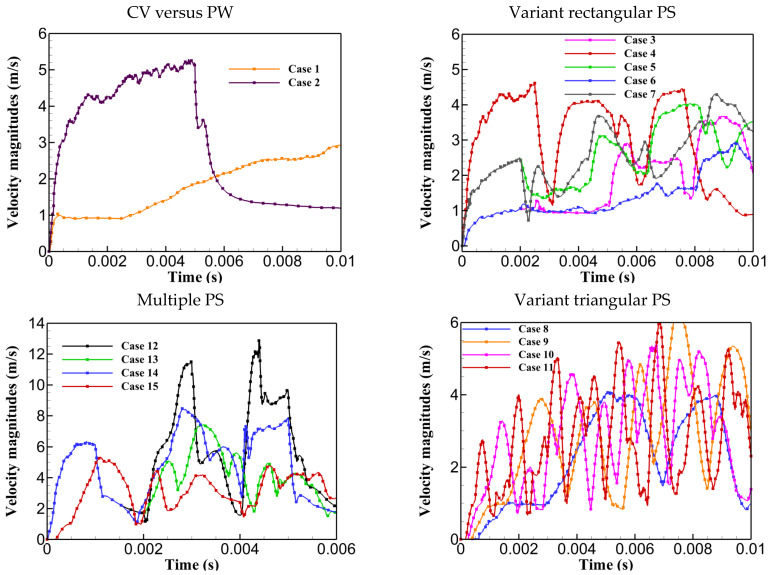
Maximum velocity magnitude for Cases 1 to 15.

This section presents the impact of pulse shaping on fluid flow behaviour, using diagrams and comparisons of velocity magnitudes for the investigated cases, as shown in Figure 7. The maximum velocity within the keyhole and molten pool correlates with the fluid flow behaviour inside these regions. High and erratic velocity magnitudes suggest strong convective currents, irregular heat and mass transfer, chaotic fluid motion, irregular keyhole walls, and higher chances of collapse. As shown in Figure 7, upon exposure to high laser energy density, followed by material fusion and evaporation, fluid velocity increased, exhibiting fluctuations in response to laser deactivation between pulses or due to specific flow dynamics and the influence of multiple forces. It was noted that PW had higher velocity magnitudes compared to CW due to the utilization of higher laser powers, which started to diminish after turning the laser off. As for the variant rectangular pulse shapes, Case 4 had the greatest velocity magnitudes with sudden spikes and falls, leading to more abrupt fluid flow behaviour within the keyhole, contributing to keyhole wall instability as corroborated in Figure 5 for keyhole morphology. Additionally, it was demonstrated that the reason for the more stable keyhole and stability in cases with single and quadruple triangular peaks compared to double and octuple triangular peaks is their smoother and lower velocity magnitudes. This led to smoother fluid flow, contributing to a more stabilized keyhole, as shown in their keyhole morphology in Figure 5. Similarly, it was further proved that the greater and more spontaneous velocity variations in Cases 12 and 14, compared to Cases 13 and 15, led to more spontaneous fluid flow, contributing to greater keyhole wall fluctuations and instability.

### 3.4. Understanding the Instability Nature of Selected Cases

**Figure 8:**
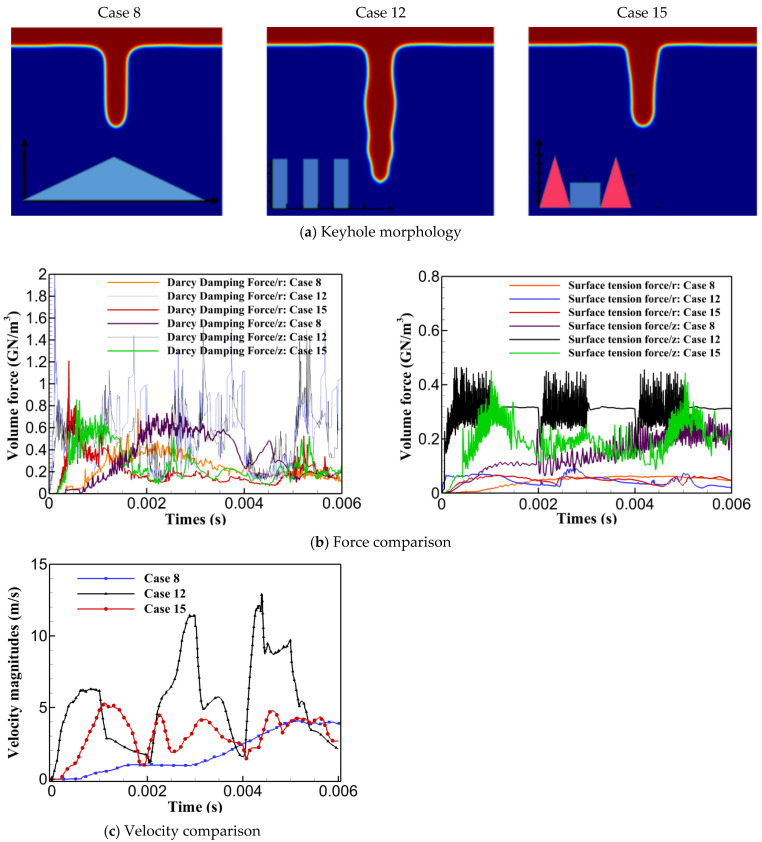
Selected cases for comprehensive and comparative instability analysis considering (**a**) keyhole morphology and (**b**,**c**) force analysis and velocity magnitude.

This section provides a comparative analysis of selected cases, considering all criteria used for instability analysis. Specifically, Case 8 and Cases 12–15 were selected to explore the correlation between keyhole stability, penetration, and process instability. Among cases with identical pulse shapes, Case 8 was chosen for its higher keyhole stability and penetration depth compared to Cases 1–11. In contrast, Cases 12–15, with multiple pulse shapes, were selected for their higher penetration depths but greater instabilities, allowing for a deeper understanding of instability behaviour and its underlying causes. Firstly, Cases 8, 12, and 15 were chosen and compared to understand the reasons behind different keyhole shapes and variant instabilities. Secondly, the impact of pulse shaping on reducing instability was analyzed to improve understanding of factors responsible for keyhole and process instability. As shown in Figure 8, Case 8 exhibited minimal keyhole instability with a smoother transition from top to bottom. In contrast, Case 12 showed significant wall fluctuations along the keyhole walls, indicating a tendency to collapse and increased instability. Case 15, however, provided a relatively shallow and wide keyhole with smoother walls, reducing the possibility of collapse. The intense instability of Case 12 was attributed to two main factors. Firstly, the utilization of high laser energy density resulted in greater Darcy’s damping force and surface tension forces in both directions compared to Cases 8 and 15. Greater Darcy’s damping force increased resistance to fluid flow, while the stronger curvature effect created a pressure difference across the keyhole walls, pulling them inward and leading to pinching, making the keyhole narrower and less stable. Secondly, Case 12 exhibited the highest and most fluctuated velocity magnitudes, indicating erratic fluid flow behaviour within the keyhole, contributing further to instability. Consequently, the combination of high-velocity magnitudes, fluctuations, pronounced curvature, Marangoni effects, and a stronger influence of Darcy’s damping force led to the increased instability observed in Case 12.

**Figure 9:**
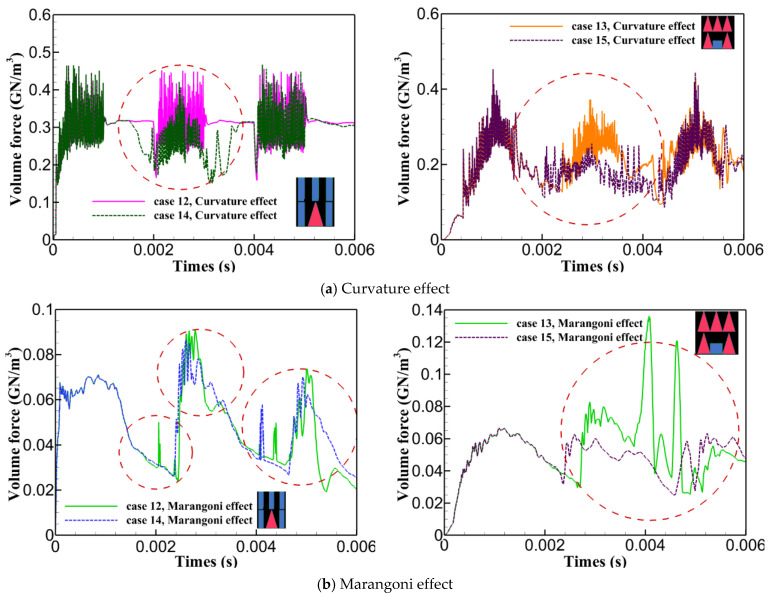
Impact of pulse shaping on instability reduction in forces for (**a**) the curvature effect and (**b**) the Marangoni effect.

Figure 9 shows the impact of using combined pulse shapes in reducing the instabilities and fluctuations in the forces, contributing to the keyhole stability. As demonstrated, using combined pulse shapes significantly reduced instability, as illustrated for Cases 12 to 15. The curvature and Marangoni effects are compared as examples to show how combined pulse shapes improved process stability by reducing force instability and fluctuations. Figure 9 depicts that using combined pulse shapes positively contributed to decreasing the instability, fluctuations, and magnitudes of the curvature effect and Marangoni effect which can be seen in the red dashed-line circle. This reduction explains the lower keyhole wall instability observed in Cases 14 and 15 compared to Cases 12 and 13. Using pulse shaping, the abrupt spikes and falls were effectively controlled and smoothed. It is also noted that a similar impact was observed for Darcy’s damping forces, although the diagrams are not provided here.

## 4. Conclusions and Future Avenues

Introducing a novel numerical approach for simulating the keyhole mode laser welding on aluminum, the impact of pulse shaping was investigated on the instability of the laser welding process. This study aims to improve the understanding of the underlying criteria contributing to the instability of the keyhole and laser welding process. The instability was comprehensively examined using analysis of the keyhole morphology, velocity magnitude and fluid flow behaviour, and instability-inducing forces such as surface tension, and Darcy’s damping force. The following conclusions are observed:

- The combination of the curvature effect, Darcy’s damping force, and more intense fluid flow behaviour contribute to the instability of the keyhole and the laser welding process.
- Using short pulse periods with higher laser energy density enhances instability and the possibility of keyhole collapse due to the increased curvature effect, Darcy’s damping force, and more intense fluid flow behaviour.
- The instability of the keyhole and process can be controlled using variant rectangular pulse shapes with gradual laser power ramp-up and -down pulse shapes due to smoother variations in velocity, smoother flow behaviour, and fewer curvature effects.
- The instability of the keyhole and forces can be controlled using combinations of triangular and rectangular pulse shapes.
- To minimize instability, the laser power should be high enough to induce evaporation and recoil pressure for keyhole propagation but balanced to avoid the excessive curvature effect, Darcy’s damping force, and fluid velocity that accompanies higher power and leads to increased instability.

Overall, this research provides key insights into the factors driving keyhole and process instability in aluminum laser welding. It quantitatively demonstrates how pulse shaping can effectively control and minimize these instabilities by influencing the forces and fluid flow behaviours involved. These findings advance understanding and offer a foundation for future research aimed at optimizing laser parameters by enhancing the understanding of the factors responsible for instability and offering strategies to control them to improve weld quality. However, some limitations remain. One key limitation is the use of a 2D axisymmetric model. While highly effective for simulating stationary spot welding and serving as a crucial first step toward more complex models, it does not fully capture the complexities of real-world laser welding scenarios that involve laser movement and multiple reflections of laser beams. Future research could explore dynamic laser welding, including the impact of multiple reflections within the keyhole on the instability and how to control and minimize the instabilities induced by multiple reflections of laser beams. This will pave the way for more advanced models with optimized welding parameters, incorporating laser movement and other real-world conditions.

## Figures and Tables

**Table 1:** Corresponding details of the case studies.

Case No.	Laser Power	Pulse Width	Total Laser Energy	Number of Pulses	Pulse Shape	Total on Time
1	2 kW	10 ms	20 J	1	Continuous	10 ms
2	4 kW	5 ms	20 J	1	Rectangular constant	5 ms
3	1–3 kW	2.5 ms	20 J	1	Rectangular: ramp up–ramp down	10 ms
4	0.5–4 kW	2.5 ms	20 J	1	Rectangular: ramp down	10 ms
5	1–3 kW	2 ms	20 J	1	Rectangular: ramp down	10 ms
6	1–3 kW	2 ms	20 J	1	Rectangular: ramp up	10 ms
7	1.5–3 kW	2 ms	20 J	1	Rectangular: ramp down-up-down	10 ms
8	0–4 kW	10 ms	20 J	1	Triangular: single peaks	10 ms
9	0–4 kW	5 ms	20 J	2	Triangular: double peaks	10 ms
10	0–4 kW	2.5 ms	20 J	4	Triangular: quadruple peaks	10 ms
11	0–4 kW	1.25 ms	20 J	8	Triangular: octuple peaks	10 ms
12	0–6 kW	1 ms	18 J	3	Rectangular	3 ms
13	0–6 kW	2 ms	18 J	3	Triangular	6 ms
14	0–6 kW	1–2 ms	18 J	3	Rectangular–triangular	4 ms
15	0–6 kW	2 ms	18 J	3	Triangular–rectangular	6 ms

**Table 2:** Thermal and physical characteristics of aluminum [35,36,37].

Property	Symbol	Magnitude
Solidus temperature	$T_{s}$	847 (K)
Liquidus temperature	$T_{l}$	905 (K)
Vaporization temperature	$T_{V}$	2743 (K)
Thermal conductivity of solid	$k_{s}$	238 (W/m/K)
Thermal conductivity of liquid	$k_{l}$	100 (W/m/K)
Density of solid	$\rho_{s}$	2700 (kg/m^3^)
Density of liquid	$\rho_{l}$	2385 (kg/m^3^)
Latent heat of melting	$L_{m}$	3.896 × 10^5^ (J/kg)
Latent heat of vaporization	$L_{V}$	9.462 × 10^6^ (J/kg)
Specific heat capacity of solid	$C_{p,s}$	917 (J/kg/K)
Specific heat capacity of liquid	$C_{p,l}$	1080 (J/kg/K)
Convective heat transfer coefficient	h	20 (W/m^2^/K)
Coefficient of linear thermal expansion	β	2.36 × 10^−5^ (1/K)
Dynamic viscosity	μ	1.6 × 10^−3^ (Pa.s)
Coefficient of surface tension	σ	0.95 × (1 + 0.13 × (1 − T/Tm))^1.67^ (N/m)
Temperature-dependent surface tension coefficient	∂σ/∂T	−0.3 × 10^−3^ (N/m/K)
Radiation emissivity	ξ	0.2

## Data Availability

The original contributions presented in the study are included in the article, further inquiries can be directed to the corresponding authors.
